# Supplementary material for: Effects of speech periodicity and speech rate on auditory-motor coupling during speech comprehension
Source: Commun Biol. 2026 Jan 8;9:205. doi: 10.1038/s42003-025-09481-y (PMC12891515; doi:10.1038/s42003-025-09481-y)
Supplement: Supplementary file 2 — Supplementary Information [file 42003_2025_9481_MOESM2_ESM.pdf]

Supplement

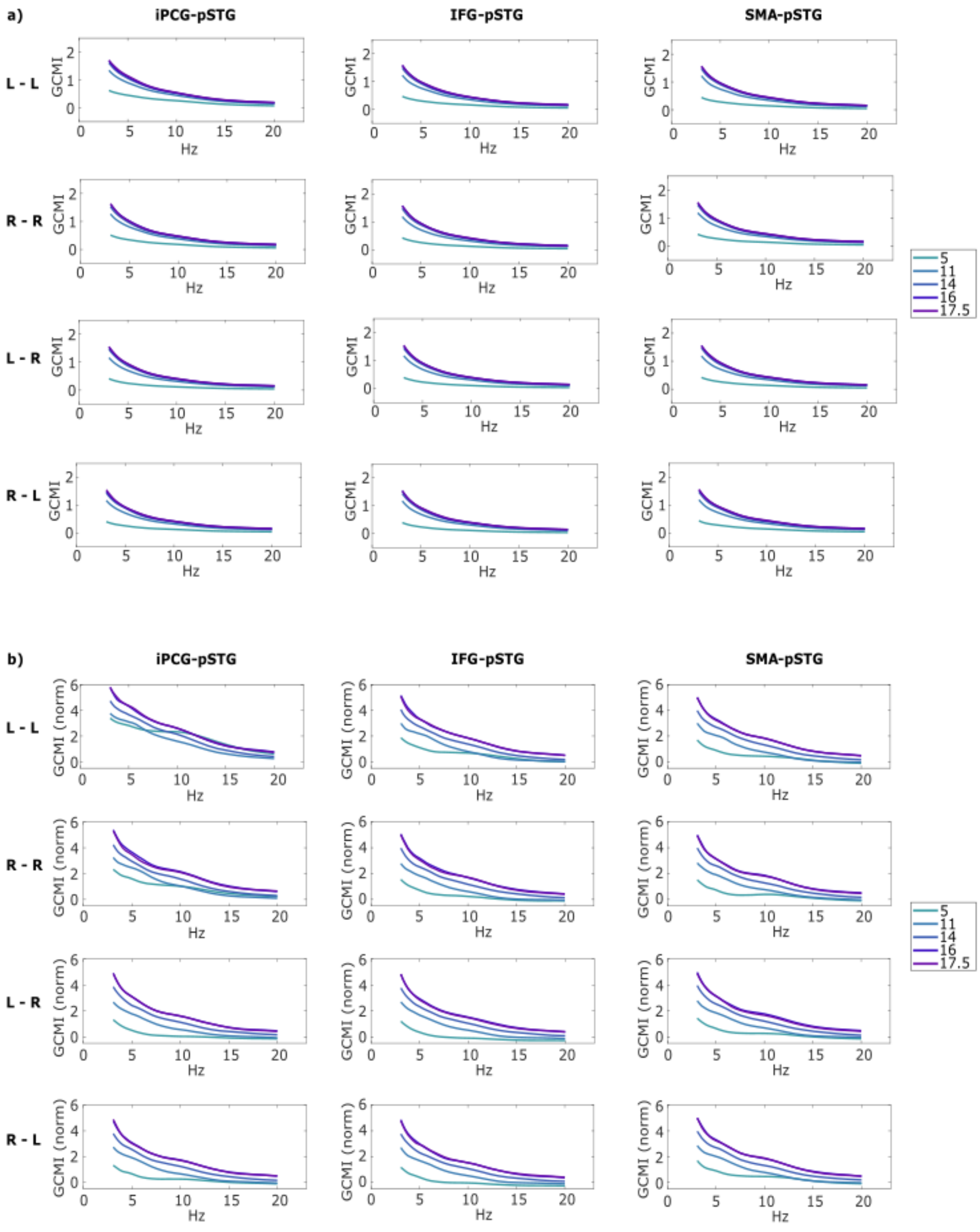

Supplementary Figure 1. The Gaussian-Copula Mutual Information (GCMI) values across frequencies are displayed for the five syllabic rate conditions. **a)** Raw GCMI values are shown across frequencies for each syllabic rate condition. **b)** Normalized GCMI values, calculated using the mean and standard deviation of surrogate data, are presented. Data is organized from left to right by ROI pairs (IPCG-pSTG, IFG-pSTG, and SMA-pSTG) and from top to bottom by hemisphere pairs (L-L, R-L, L-R, and R-R).

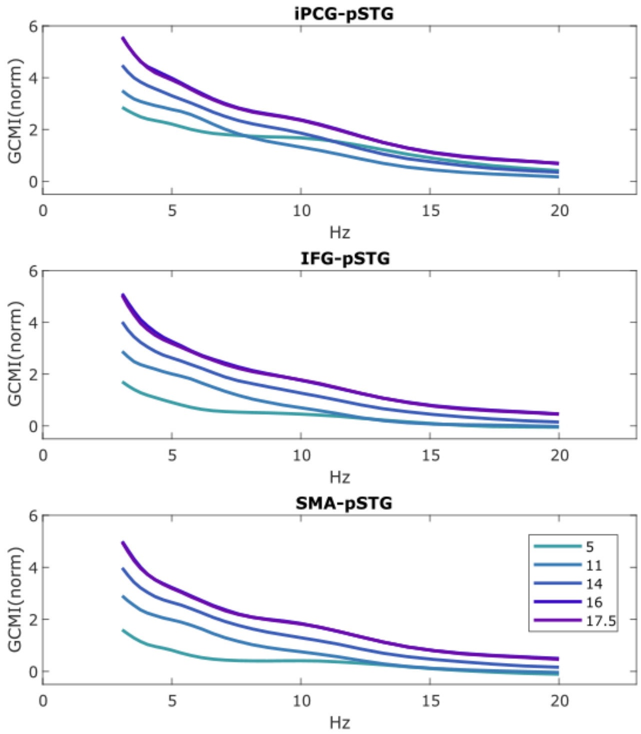

Supplementary Figure 2. The average Gaussian-Copula Mutual Information (GCMI) for ipsilateral ROI pairs across syllabic rate conditions is displayed. IPCG-pSTG, IFG-pSTG, and SMA-pSTG from top to bottom. The theta frequency range (4-8Hz) was selected for subsequent analyses.

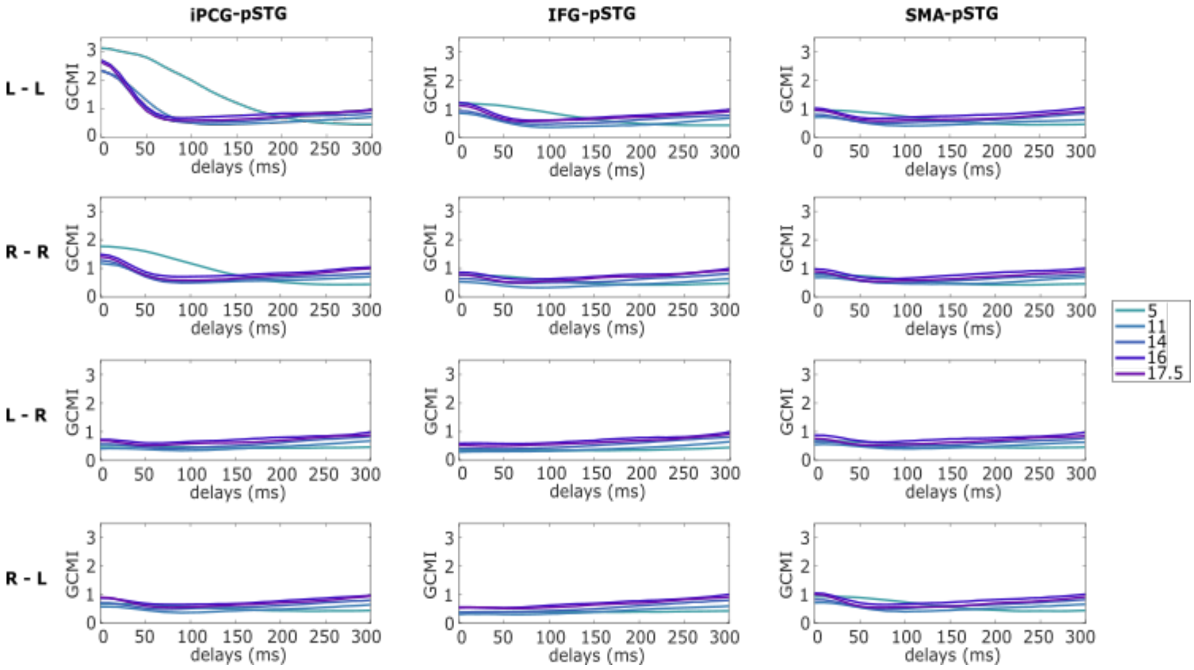

Supplementary Figure 3. This figure shows Gaussian-Copula Mutual Information (GCMI) values across delays from 0ms to 300ms, calculated in 10ms intervals for all ROI pairs. The highest MI occurred at 0ms, followed by a plateau until 50ms, after which values declined. A 50ms delay, marking the end of the plateau, was selected for further analysis to balance MI optimization and physiological plausibility. The plot displays average GCMI values for all participants and brain region pairs, grouped by syllabic rate. Columns correspond to specific ROI pairs, while rows indicate hemispheric pairing (L-L, R-R, L-R, R-L, from top to bottom).

**Predictors**	**Estimates**	**CI (95%)**	**Z-values**	**p-values (FDR adjusted)**
(Intercept)	2.30	(1.72, 2.88)	7.81	**1.49×10^{-14}**
Syllabic rate	-0.16	(-0.21, -0.12)	-6.94	**0.005**
MAD	3.41	(2.42, 4.41)	6.73	**2.30×10^{-11}**
Syllabus number	-0.38	(-0.46, -0.29)	-8.00	**3.15×10^{-17}**
Stimulation order	0.09	(0.07, 0.10)	10.70	**8.45×10^{-26}**
Sentence-level average word-frequency	0.02	(-0.07, 0.10)	0.38	0.703
Compression	0.22	(0.16, 0.28)	7.00	**5.23×10^{-12}**
Syllabic rate x MAD	-0.21	(-0.29, -0.13)	-4.96	**8.11×10^{-7}**
**Random Effects**				

σ^2	0.00
τ_00 Trials	1.07
τ_00 Participants.1	0.58
τ_11 Participants.Syllabic rate	0.003
ICC	1.00
N Participants	57
N Trials	579
Observation	17100
Marginal R^2 / Conditional R^2	0.503/0.999

Supplementary Table 1. Comprehensive statistics of the analysis of the effects of periodicity on speech comprehension performance, including the results for the fixed effects and the interaction effect between periodicity and syllabic rate. The GLMM model is fitted with TMB using a beta family. The equation for this model is: Speech comprehension performance ~ syllabic rate * periodicity + compression factor + sentence-level average word-frequency + syllabus number + stimulation order + (1| trials) + (1|participants) + (0 + syllabic rate | subject).

**Predictors**	**Estimates**	**CI (95%)**	**Z-values**	**p-values (FDR adjusted)**
(Intercept)	-1.51	(-1.52, -1.50)	-261.61	**0×10^{-0}**
Syllabic rate	151.30	(145.81, 156.79)	54.02	**0×10^{-0}**
Syllabic rate^2	15.22	(9.16, 21.28)	4.92	**1.93×10^{-6}**
Syllabic rate^3	10.26	(3.92, 16.59)	3.18	**0.002**
MAD	0.10	(0.09, 0.11)	13.76	**1.71×10^{-42}**
Brain_area iPCG-pSTG	0.16	(0.15, 0.16)	34.31	**4.17×10^{-257}**

Brain_area SMA-pSTG	-0.01	(-0.02, -0.01)	-3.17	**0.002**
Sentence-level average word-frequency	-0.004	(-0.01, -0.004)	-8.43	**1.01 x 10^{-16}**
Hemisphere_R	-0.09	(-0.09, -0.08)	-77.46	**0 x 10^{-0}**
Compression	0.01	(0.01, 0.01)	16.11	**1.13 x 10^{-10}**
Syllabic rate x MAD	37.70	(25.94, 49.45)	6.29	**8.01 x 10^{-10}**
Syllabic rate^2 x MAD	-43.98	(-57.21, -30.74)	-6.51	**1.98 x 10^{-10}**
Syllabic rate^3 x MAD	-62.39	(-76.01, -48.16)	-8.74	**8.05 x 10^{-18}**
Syllabic rate x Brain_area iPCG-pSTG	-59.16	(-66.65, -51.66)	-15.47	**2.48 x 10^{-53}**
Syllabic rate^2 x Brain_area iPCG-pSTG	12.33	(4.00, 20.65)	2.90	**0.005**
Syllabic rate^3 x Brain_area iPCG-pSTG	5.83	(-2.93, 14.59)	1.30	0.236
Syllabic rate x Brain_area SMA-pSTG	8.14	(0.43, 15.84)	2.07	**0.049**
Syllabic rate^2 x Brain_area SMA-pSTG	-9.34	(-17.85, -0.83)	-2.15	**0.042**
Syllabic rate^3 x Brain_area SMA-pSTG	-17.66	(-26.60, -8.73)	-3.87	**5.87 x 10^{-2}**
MAD:Brain_area iPCG-pSTG	0.02	(0.00, 0.04)	2.24	**0.035**
MAD:Brain_area SMA-pSTG	0.01	(-0.01, 0.03)	1.15	0.293
Syllabic rate x MAD x Brain_area iPCG-pSTG	3.05	(-13.10, 19.21)	0.37	0.738
Syllabic rate^2 x MAD x Brain_area iPCG-pSTG	24.03	(5.83, 42.24)	2.59	**0.014**
Syllabic rate^3 x MAD x Brain_area	-10.72	(-29.99, 8.55)	-1.09	0.371

iPCG-pSTG				
Syllabic rate x MAD x Brain_area SMA-pSTG	0.30	(-16.30, 16.90)	0.04	0.965
Syllabic rate^2 x MAD x Brain_area SMA-pSTG	10.17	(-8.44, 28.78)	1.07	0.306
Syllabic rate^3 x MAD x Brain_area SMA-pSTG	44.73	(25.07, 64.39)	4.46	**1.71 x 10^{-5}**
**Random Effects**				
σ^2	0.17			
τ_00 Participants	0.001			
τ_00 Trials	0.0007			
ICC	0.01			
N Participants	57			
N Trials	300			
Observation	905880			
Marginal R^2 / Conditional R^2	0.161/0.170			

Supplementary Table 2. Comprehensive statistics of the analysis of the effect of periodicity on neural coupling strength (GCMI), including the results for the fixed effects and interaction effects. These include interactions such as syllabic rate × MAD, MAD × ROI pairs, and ROI pairs × syllabic rate, as well as the three-way interaction among syllabic rate, MAD, and ROI pairs. For this model, GLMM is fitted with a TMB using the beta family. The model equation is: Mutual information ~ syllabic rate^3 * periodicity * Region of interest + compression factor + sentence-level average word-frequency + hemisphere + (1| trials) + (1| subject).

**Predictors**	**Estimates**	**CI (95%)**	**Z-values**	**p-values (FDR adjusted)**
(Intercept)	2.44	(2.30, 2.57)	35.47	**4.23 x 10^{-275}**
Syllabic rate	-0.17	(-0.17, -0.16)	-86.18	**0 x 10^{-0}**

GCMI	3.46	(3.18, 3.75)	23.77	**1.33 x 10^{-124}**
MAD	0.31	(0.22, 0.41)	6.37	**2.06 x 10^{-10}**
Sentence-level average word-frequency	0.03	(0.03, 0.03)	24.74	**1.09 x 10^{-134}**
Compression	0.14	(0.14, 0.14)	98.30	**0 x 10^{-0}**
Syllabic rate x GCMI	-0.25	(-0.27, -0.23)	-23.48	**1.01 x 10^{-121}**
Syllabic rate x MAD	0.02	(0.01, 0.03)	4.41	**1.03 x 10^{-5}**
GCMI x MAD	-3.36	(-3.96, -2.76)	-11.0	**5.39 x 10^{-28}**
Syllabic rate x MAD x GCMI	0.19	(0.15, 0.24)	8.49	**2.67 x 10^{-17}**
**Random Effects**				
σ^2	0.981			
τ_00 Participants	0.23			
τ_00 Trials	0.05			
ICC	0.49			
N Participants	57			
N Trials	300			
Observation	905880			
Marginal R^2 / Conditional R^2	0.585/0.790			

Supplementary Table 3. Comprehensive statistics of the direct analysis of the speech comprehension – neural coupling relationship. Speech comprehension was predicted by the neural coupling, the periodicity (MAD), the syllabic rate and their interactions using GLMM. Model fitting was performed using the TMB, specifically with the beta family. The used model equation is: Speech comprehension performance ~ GCMI * syllabic rate * periodicity + compression factor + sentence-level average word-frequency + syllabus number + stimulation order + (1| trials) + (1|participants).
