# Supplementary material for: Effects of speech periodicity and speech rate on auditory-motor coupling during speech comprehension
Source: Commun Biol. 2026 Jan 8;9:205. doi: 10.1038/s42003-025-09481-y (PMC12891515; doi:10.1038/s42003-025-09481-y)
Supplement: Supplementary file 3 — reporting summary [file 42003_2025_9481_MOESM3_ESM.pdf]

nature portfolio

Corresponding author(s): Johanna M. Rimmele

Last updated by author(s): 12.02.2025

Reporting Summary

Nature Portfolio wishes to improve the reproducibility of the work that we publish. This form provides structure for consistency and transparency in reporting. For further information on Nature Portfolio policies, see our Editorial Policies and the Editorial Policy Checklist.

Statistics

For all statistical analyses, confirm that the following items are present in the figure legend, table legend, main text, or Methods section.

n/a	Confirmed	
☐	☒	The exact sample size (*n*) for each experimental group/condition, given as a discrete number and unit of measurement
☒	☐	A statement on whether measurements were taken from distinct samples or whether the same sample was measured repeatedly
☐	☒	The statistical test(s) used AND whether they are one- or two-sided<br>*Only common tests should be described solely by name; describe more complex techniques in the Methods section.*
☐	☒	A description of all covariates tested
☐	☒	A description of any assumptions or corrections, such as tests of normality and adjustment for multiple comparisons
☐	☒	A full description of the statistical parameters including central tendency (e.g. means) or other basic estimates (e.g. regression coefficient) AND variation (e.g. standard deviation) or associated estimates of uncertainty (e.g. confidence intervals)
☒	☐	For null hypothesis testing, the test statistic (e.g. *F*, *t*, *r*) with confidence intervals, effect sizes, degrees of freedom and *P* value noted<br>*Give P values as exact values whenever suitable.*
☒	☐	For Bayesian analysis, information on the choice of priors and Markov chain Monte Carlo settings
☐	☒	For hierarchical and complex designs, identification of the appropriate level for tests and full reporting of outcomes
☒	☐	Estimates of effect sizes (e.g. Cohen's *d*, Pearson's *r*), indicating how they were calculated

Our web collection on statistics for biologists contains articles on many of the points above.

Software and code

Policy information about availability of computer code

Data collection	A statement is included in the manuscript: During each trial, a sentence was presented through EARTONE Gold 3A earplugs, controlled by Matlab (R2017a) with the Psychtoolbox extension 90 on a Fujitsu-Technology CELSIUS R940power PC.
Data analysis	All custom code central to the conclusions will be made available upon acceptance in a public repository.

For manuscripts utilizing custom algorithms or software that are central to the research but not yet described in published literature, software must be made available to editors and reviewers. We strongly encourage code deposition in a community repository (e.g. GitHub). See the Nature Portfolio guidelines for submitting code & software for further information.

Data

Policy information about availability of data

All manuscripts must include a data availability statement. This statement should provide the following information, where applicable:

- Accession codes, unique identifiers, or web links for publicly available datasets
- A description of any restrictions on data availability
- For clinical datasets or third party data, please ensure that the statement adheres to our policy

The anonymized preprocessed MEG and behavioral data will be made available upon acceptance in the context of Lubinus et al. 91 The link to the repository will be provided. Due to restrictions the pseudonymized raw MRI and MEG data, as well as the unprocessed stimulus material is not publicly available.

Research involving human participants, their data, or biological material

Policy information about studies with human participants or human data. See also policy information about sex, gender (identity/presentation), and sexual orientation and race, ethnicity and racism.

Reporting on sex and gender	The study analyzed data from a final sample of 57 participants91, all of whom completed every condition of the experiment under the same procedure. Initially, 60 individuals (age: M = 26.9, SD = 5.4 years; 32 females, 28 males, based on self-reported gender) were recruited from the MPI database, drawing from the local Frankfurt community.
Reporting on race, ethnicity, or other socially relevant groupings	No reporting on race, ethicity was conducted. Other socially relevant groupings were the following: "All participants confirmed the absence of neurological or psychiatric disorders in a self-report assessment. Additionally, participants were required to have normal hearing and normal or corrected-to-normal vision. All participants were native German speakers and right-handed."
Population characteristics	The study analyzed data from a final sample of 57 participants91, all of whom completed every condition of the experiment under the same procedure. Initially, 60 individuals (age: M = 26.9 years, SD = 5.4 years; 32 females, 28 males, based on self-reported gender) were recruited from the MPI database, drawing from the local Frankfurt community. However, three participants were excluded due to technical issues during recording or due to an average performance that was three standard deviations below the mean in the baseline speech comprehension task (5 syllables/s) 91. All participants confirmed the absence of neurological or psychiatric disorders in a self-report assessment. Additionally, participants were required to have normal hearing and normal or corrected-to-normal vision. All participants were native German speakers and right-handed, factors which were considered to maintain consistency and minimize potential confounding variables across the study population.
Recruitment	The participants were recruited from the data base of the Max Planck Insitute for empirical Aesthetics following the requirements stated above
Ethics oversight	The study91 was approved by the local ethics committee of the University Hospital of the Goethe-University Frankfurt (approval number: 2021-509) and conducted in accordance with the Declaration of Helsinki. All participants gave informed consent to the participation.

Note that full information on the approval of the study protocol must also be provided in the manuscript.

Field-specific reporting

Please select the one below that is the best fit for your research. If you are not sure, read the appropriate sections before making your selection.

☒ Life sciences ☐ Behavioural & social sciences ☐ Ecological, evolutionary & environmental sciences

For a reference copy of the document with all sections, see nature.com/documents/nr-reporting-summary-flat.pdf

Life sciences study design

All studies must disclose on these points even when the disclosure is negative.

Sample size	The data were recorded for a previous study (Lubinus, Keitel, Obleser, Poeppel, Rimmele, 2025, BioRxiv). In this context the sample size was based on a previous study that investigated effects of auditory-motor synchronization on speech processing (Assaneo, Rimmele et al., 2021, Nature Human behavior). The study included Monte Carlo Simulation estimating the probability of false evidence.
Data exclusions	The initial sample included n=60 participants. Three participants had to be excluded due to technical issues with data recording
Replication	This study includes no replication study.
Randomization	Participants listened to spoken sentences. The presentation order of sentence was randomized differently for each participant
Blinding	There was no blinding, as no group allocation was made.

Reporting for specific materials, systems and methods

We require information from authors about some types of materials, experimental systems and methods used in many studies. Here, indicate whether each material, system or method listed is relevant to your study. If you are not sure if a list item applies to your research, read the appropriate section before selecting a response.

Materials & experimental systems

n/a	Involved in the study
☒	☐ Antibodies
☒	☐ Eukaryotic cell lines
☒	☐ Palaeontology and archaeology
☒	☐ Animals and other organisms
☒	☐ Clinical data
☒	☐ Dual use research of concern
☒	☐ Plants

Methods

n/a	Involved in the study
☒	☐ ChIP-seq
☒	☐ Flow cytometry
☐	☒ MRI-based neuroimaging

Plants

Seed stocks

n/a

Novel plant genotypes

n/a

Authentication

n/a

Magnetic resonance imaging

Experimental design

Design type

individual structural MRI scans were acquired to inform MEG source imaging; recorded while the participant was resting

Design specifications

n/a

Behavioral performance measures

n/a

Acquisition

Imaging type(s)

structural

Field strength

3 Tesla

Sequence & imaging parameters

Imaging type: T1-weighted scan
RF Pulse type: Normal
Matrix size: Voxel size: 1.0×1.0×1.0 mm
Slice thickness: 1 mm
Flip angle: 8 deg;
TR/TE: 2300/2.28
Initial Orientation: S>T

Area of acquisition

whole brain scan

Diffusion MRI ☐ Used ☒ Not used

Preprocessing

Preprocessing software

Fieldtrip toolbox (version 20221223, Oostenveld et al., 2011), using SPM12
To align the MRI and MEG data for source reconstruction, Vitamin E capsules were placed at key anatomical landmarks: the nasion and the left and right preauricular points. These landmarks were used to co-register the MRI scans with the MEG coordinate system through a semi-automated process.

Normalization

T1-weighted MRIs were segmented into white matter, gray matter, and cerebrospinal fluid to create single-shell volume conduction models (head models), following the method outlined by Nolte et al. (2008). These images were normalized to MNI space, and individual grids with a resolution of 5 mm were generated by inverse-warping a template grid to align with each participant's anatomical structure. Using the individual grids and volume conduction models, forward models were computed to reconstruct source activity. Source reconstruction was performed using the Linearly Constrained Minimum

Variance (LCMV) Beamformer method, as described by Westner et al. (2022).

Normalization template: the SPM T1 template integrated in the fieldtrip toolbox was used to make a template grid

Noise and artifact removal: Noise and artifact removal was only performed for the MEG data, not the structural MRI data

Volume censoring: n/a

Statistical modeling & inference

Model type and settings: Our analysis were performed on the MEG source space data, no analysis were performed on the structural MRI data. We used Gaussian-Copula Mutual Information (GCMI) to quantify the MEG phase-phase coupling between auditory and speech motor areas in the theta frequency band. Linear mixed-effects model (LMM) where used to predict the neural coupling (MEG) based on speech periodicity and other predictors

Effect(s) tested: Linear mixed-effects model (LMM) where used to predict the neural coupling (MEG) based on speech periodicity (median absolute deviation between syllable nuclei) and other predictors

Specify type of analysis: ☐ Whole brain ☒ ROI-based ☐ Both

Anatomical location(s): Automated Anatomical Labeling (AAL) atlas-defined regions of interest (ROI) were utilized. Original AAL ROIs' are 1 and 2 for PCG (right and left, AAL label: Precentral), 13 and 14 for IFG (right and left, AAL label: Frontal_Inf_Tri), 19 and 20 for SMA (right and left, AAL label: Supp_Motor_Area), and 81 and 82 for STG (superior temporal gyrus, right and left, AAL label: Temporal_Sup). Among these, PCG and STG underwent additional division in order to have inferior parts of PCG (iPCG) and posterior parts of STG (pSTG). PCG was divided horizontally into three equal parts at the one-third and two-thirds marks, and STG was divided in half along the vertical midline of the voxel. We focused on the iPCG because it corresponds to the tongue and face regions, which are particularly important for language processing (96).

Statistic type for inference (See Eklund et al. 2016): The variable ROI was used as fixed effect in the generalized linear mixed models (GLMM)

Correction: FDR correction was applied to the generalized linear mixed models (GLMM) and all post hoc tests

Models & analysis

n/a	Involved in the study
☐	☒ Functional and/or effective connectivity
☒	☐ Graph analysis
☐	☒ Multivariate modeling or predictive analysis

Functional and/or effective connectivity: Gaussian-Copula mutual information (GCMI) was used to quantify functional connectivity as phase-phase coupling

Multivariate modeling and predictive analysis: e behavioral data (n=57) were analyzed using generalized linear mixed models (GLMM). Model fitting was performed using the Template Model Builder (TMB) in R, specifying the beta family. All analyses used the glmmTMB package with a beta distribution to appropriately model sentence-wise data bounded between 0 and 1. To address boundary values at No multicollinearity issues were observed.

Sentence-wise speech comprehension performance (measured as % correct) was modeled as a function of syllabic rate, periodicity, compression factor, syllable number, stimulation order, and sentence-level word complexity. The word complexity was estimated based on the average word frequency data per sentence from the Leipziger Wortschatz Corpus (Mixed-Typical 2011 dataset). The model included an interaction term between syllabic rate and periodicity to examine their combined influence on comprehension. Random effects were included to account for variability across participants and audio files. This model structure allowed us to capture both fixed and random effects while addressing variability inherent to the experimental design.

For the statistical analysis of GCMI, generalized linear mixed models (GLMM) were employed (n=57). Specifically, the template model builder (TMB) function in the R statistical software package was used for model fitting specifying the beta family to address heteroscedasticity issues. Prior to the analysis the normalized GCMI data were scaled and min-max normalized to meet the beta distribution requirements, with data bounded between 0 and 1. Boundary values at 0 and 1, were adjusted by adding or subtracting a small value, calculated as ± (median/sample size). No multicollinearity issues were observed.

For the neural model, mutual information (GCMI) was analyzed as a function of syllabic rate, periodicity, ROI and hemisphere. The model incorporated interaction terms between syllabic rate, periodicity, and ROI and hemisphere and periodicity to assess how these factors jointly influence neural measures. Additionally, compression factor and sentence-averaged word complexity were included as covariates to control for their potential confounding effects. Random intercepts were included for both trials and participants to account for variability at each level, providing a robust framework to capture both within-subject (trial-level) and

between-subject variation. A third-degree polynomial (poly(3)) was selected based on the data distribution, its better model performance, and generalization capabilities.
